# Supplementary material for: Hydrogen and deuterium charging of lifted-out specimens for atom probe tomography
Source: Open Res Eur. 2022 Feb 21;1:122. Originally published 2021 Oct 14. [Version 2] doi: 10.12688/openreseurope.14176.2 (PMC10445872; doi:10.12688/openreseurope.14176.2)
Supplement: Peer review history

# Peer review: Reviewer response for version 2

**DOI:** 10.21956/openreseurope.15707.r28637

**Published:** 2022-03-07

**Authors:** Gregory Thompson

**Affiliations:** 1 Department of Metallurgical and Materials Engineering, University of Alabama, Tuscaloosa, AL, USA

The authors have addressed the prior concerns. I approve the new version of this article.

Is the rationale for developing the new method (or application) clearly explained?

Partly

Is the description of the method technically sound?

Yes

Are the conclusions about the method and its performance adequately supported by the findings presented in the article?

Yes

If any results are presented, are all the source data underlying the results available to ensure full reproducibility?

Partly

Are sufficient details provided to allow replication of the method development and its use by others?

Yes

Reviewer Expertise:

atom probe. TEM, materials processing, nanocrystalline metals.

I confirm that I have read this submission and believe that I have an appropriate level of expertise to confirm that it is of an acceptable scientific standard.

# Peer review: Reviewer response for version 2

**DOI:** 10.21956/openreseurope.15707.r28636

**Published:** 2022-03-07

**Authors:** Maria Auger

**Affiliations:** 1 Department of Materials, University of Oxford, Oxford, UK

I thank the authors for considering and answering my questions.

Is the rationale for developing the new method (or application) clearly explained?

Yes

Is the description of the method technically sound?

Yes

Are the conclusions about the method and its performance adequately supported by the findings presented in the article?

Yes

If any results are presented, are all the source data underlying the results available to ensure full reproducibility?

Yes

Are sufficient details provided to allow replication of the method development and its use by others?

Partly

Reviewer Expertise:

atom probe tomography; APT sample preparation; FIB; SEM; metallurgy; microstructural characterization; mechanical characterization;

I confirm that I have read this submission and believe that I have an appropriate level of expertise to confirm that it is of an acceptable scientific standard.

# Peer review: Reviewer response for version 1

**DOI:** 10.21956/openreseurope.15283.r27926

**Published:** 2021-11-17

**Authors:** Gregory Thompson

**Affiliations:** 1 Department of Metallurgical and Materials Engineering, University of Alabama, Tuscaloosa, AL, USA

This paper provides an overview of different specimen preparation methods to study deuterium/hydrogen by atom probe. In large part, the work reported has been done by many other authors with this manuscript being a collection of those techniques. The reviewer does recognize that these other works largely focused on electro-polishing samples while this work addresses FIB preparation. The outcomes, in many cases, were the same between this work and the prior reports. These similar outcomes include delamination (Haley *et al.*), ‘frost/ice’ concerns (Chen *et al.*), plasma charging, sulfuric acid, and gas charging (Khanchandani *et al.*). The authors correctly note that their unsuccessful use of a plasma charge is likely tied to the low vacuum levels and not the method. They ultimately conclude, as did the prior work, to proceed is best used with gas charging (WF7) and electro-charging (WF5).

 In many cases, the referencing to this prior work is provided; however, the authors could offer further clarity by specifically citing the outcomes of their work to that of the others or expanding that discussion. For example, in the gas charging section (WF7), the conditions of the experiment are given but no rational on why they were chosen. Their parameters appear similar to Takahashi *et al.* 2010 paper.  A Takahashi *et al.* reference is provided (#15, 2018), but is not connected to the gas charge experimental section. 

 The online version of the manuscript places Fig 1 (the overall workflow plan) well ahead of any of the text regarding the workflows. This made its initial presentation difficult to understand or integrate to the reading. The authors may want to consider this figure as a summary description of the workflows after each is presented, since the figure’s context contains all the information that is yet to be presented or understood by the reader.

 Since the novelty of this work is the site-specific preparation, the paper could be enhanced by focusing more of its discussion and conclusion about these advantages and disadvantages for a FIB prepared sample, specifically noting that the  title states ‘site specific specimen(s).’ In the current format, the paper reads as a review of using prior methods to a generally prepared FIB sample. 

 The reviewer appreciates the details on the use of Mo grids rather than Cu in the electro-charging. Such information is very useful to new users that may overlook such issues about re-deposition/dissolution.

 The paper would be benefited by further explanation to why a FIB mill after exposure to the deuterium/hydrogen environment was needed as a clean up and how that may affect the results. Often the APT  sample is run prior to charging to establish a base line mass spectrum after which it is charged and run immediately to see the change as noted in the gas charging experiment (WF7). The authors correctly note that a post FIB mill after a charge would alter such a comparison but why it (i.e., the post-mill) was even needed was unclear. Is it an issue with FIB samples compared to electropolished samples? Further discussions regarding the post-charge FIB (it advantages and disadvantages) would be informative for users who are considering this method of preparation.  

 The reviewer appreciates the discussion regarding charge state ratios and its relationship to both different atom probe instruments as well as charging techniques. This information will be informative for future readers.

Is the rationale for developing the new method (or application) clearly explained?

Partly

Is the description of the method technically sound?

Yes

Are the conclusions about the method and its performance adequately supported by the findings presented in the article?

Yes

If any results are presented, are all the source data underlying the results available to ensure full reproducibility?

Partly

Are sufficient details provided to allow replication of the method development and its use by others?

Yes

Reviewer Expertise:

atom probe. TEM, materials processing, nanocrystalline metals.

I confirm that I have read this submission and believe that I have an appropriate level of expertise to confirm that it is of an acceptable scientific standard, however I have significant reservations, as outlined above.

# Response

**Published:** 2022-02-17

**Authors:** Heena Khanchandani

**Affiliations:** Max Planck Institute for Iron Research Dusseldorf, Germany, Germany

Thank you so much for your valuable comments which were helpful in improving the manuscript. Your individual comments are addressed in italics as follows:

**Comment: **In many cases, the referencing to this prior work is provided; however, the authors could offer further clarity by specifically citing the outcomes of their work to that of the others or expanding that discussion. For example, in the gas charging section (WF7), the conditions of the experiment are given but no rational on why they were chosen. Their parameters appear similar to Takahashi *et al.* 2010 paper. A Takahashi *et al.* reference is provided (#15, 2018), but is not connected to the gas charge experimental section.

***Reply:*** *Thanks for pointing this out. The same reference to Jun Takahashi et al.’s 2018 paper has now been connected to the gas charging experimental section where gas charging parameters are mentioned.*

**Comment: **The online version of the manuscript places Fig 1 (the overall workflow plan) well ahead of any of the text regarding the workflows. This made its initial presentation difficult to understand or integrate to the reading. The authors may want to consider this figure as a summary description of the workflows after each is presented, since the figure’s context contains all the information that is yet to be presented or understood by the reader.

***Reply: **Figure 1 has now been moved to right before the description of all workflows, as Figure 4. It serves as an introduction to all the workflows that subsequently follow in the manuscript*

**Comment: **Since the novelty of this work is the site-specific preparation, the paper could be enhanced by focusing more of its discussion and conclusion about these advantages and disadvantages for a FIB prepared sample, specifically noting that the  title states ‘site specific specimen(s).’ In the current format, the paper reads as a review of using prior methods to a generally prepared FIB sample.

***Reply:*** *Those aspects have been discussed in the introduction section in the original version, but are now included at the beginning of the Results and discussion section as well.* *The title of the manuscript has also been modified to emphasize that the work is focused on the lifted-out specimens.*

**Comment:**The reviewer appreciates the details on the use of Mo grids rather than Cu in the electro-charging. Such information is very useful to new users that may overlook such issues about re-deposition/dissolution.

 The paper would be benefited by further explanation to why a FIB mill after exposure to the deuterium/hydrogen environment was needed as a clean up and how that may affect the results. Often the APT  sample is run prior to charging to establish a base line mass spectrum after which it is charged and run immediately to see the change as noted in the gas charging experiment (WF7). The authors correctly note that a post FIB mill after a charge would alter such a comparison but why it (i.e., the post-mill) was even needed was unclear. Is it an issue with FIB samples compared to electropolished samples? Further discussions regarding the post-charge FIB (it advantages and disadvantages) would be informative for users who are considering this method of preparation.

***Reply: **A further clarification to this point has now been added in the Results and discussion section.* *Also, as shown in Figure 8, there was often frost on the APT tip after charging, despite the UHV transfer, which needed to be cleaned by FIB mill before analysis, otherwise, the specimens would not run.*

# Peer review: Reviewer response for version 1

**DOI:** 10.21956/openreseurope.15283.r27812

**Published:** 2021-11-08

**Authors:** Maria Auger

**Affiliations:** 1 Department of Materials, University of Oxford, Oxford, UK

This paper is very well written, it can be easily followed and understood, the list of references is adequate and the underlying data are available in the corresponding link. It provides insightful experimental details for charging site-specific APT specimens with hydrogen/deuterium through 3 different routes and following 7 different workflows. Each route is explained in high detail, with both the figures and images helping to visualize the described configurations. The success or not obtained in each workflow is correctly shown and explained. From an experimental point of view, it is really helpful to identify when something does not go as expected. In this specific case, it is very useful to have images of how moisture or frozen ethanol look like on the sample or how a damaged feature displays during FIB sample preparation.

 My answer to the question 'Are sufficient details provided to allow replication of the method development and its use by others?' was 'Partly'. The reason for this is because the results discussed in this paper are specific to the TWIP steel used as reference material. This fact is not mentioned in the title, the abstract or the conclusions, where the work is described as facilitating the study of hydrogen in metals, being easy to think that the work may refer to metals in general. The successful routes described in this work and the experimental parameters used here might not work in the same way for other materials (W, metallic multilayers, Zr alloys, Ti alloys, etc.) susceptible to hydrogen embrittlement as well.

 My 'Partly' answer to this question is also due to the very specific framework (Laplace project) used to conduct the study. For somebody else willing to replicate the method, they would have to build a similar framework (not easy...), ask for access to this facility or try accessing a similar facility somewhere else, for example the one described in reference [1] below or some of those mentioned in reference 26 of the manuscript.

 I found the first paragraph of the Introduction a bit vague compared to the rest of the Introduction or the rest of the paper. The different statements didn't seem to be connected or, at least, that was my perception.

 I could spot 2 or 3 minor typos and 3 reference links not displayed correctly:

 Link to reference 1 not included, can be found as in [2]

 Reference 5 not displayed correctly, might be as [3]

 Link to reference 49 not displayed, might be as [4]

 The description of the thickness 't' in Figure 4 is not clear... Is it correctly located in Fig. 4(a)? The 3um and 1um values mentioned in captions (b), (c) and (d), do they correspond to different samples? or to different stages in the same sample preparation process? It is a bit confusing.

 This manuscript can be indexed with minor corrections. I hope that my comments might help to improve this already very good work.

Is the rationale for developing the new method (or application) clearly explained?

Yes

Is the description of the method technically sound?

Yes

Are the conclusions about the method and its performance adequately supported by the findings presented in the article?

Yes

If any results are presented, are all the source data underlying the results available to ensure full reproducibility?

Yes

Are sufficient details provided to allow replication of the method development and its use by others?

Partly

Reviewer Expertise:

atom probe tomography; APT sample preparation; FIB; SEM; metallurgy; microstructural characterization; mechanical characterization;

I confirm that I have read this submission and believe that I have an appropriate level of expertise to confirm that it is of an acceptable scientific standard.

## References

[1] : A versatile cryo-transfer system, connecting cryogenic focused ion beam sample preparation to atom probe microscopy. *PLoS One* .2021;16(1) : 10.1371/journal.pone.0245555 e0245555 10.1371/journal.pone.0245555 33465106PMC7815152

[2] : FIM/IAP/TEM STUDIES OF HYDROGEN IN METALS. *Le Journal de Physique Colloques* .1984;45(C9) : 10.1051/jphyscol:1984959 C9-360 10.1051/jphyscol:1984959

[3] : Prevention of Hydrogen Embrittlement in Steels. *ISIJ International* .2016;56(1) : 10.2355/isijinternational.ISIJINT-2015-430 24-36 10.2355/isijinternational.ISIJINT-2015-430

[4] : The hydrogen content of austenite after cathodic charging. *Scripta Metallurgica* .1981;15(6) : 10.1016/0036-9748(81)90047-8 661-664 10.1016/0036-9748(81)90047-8

# Response

**Published:** 2022-02-17

**Authors:** Heena Khanchandani

**Affiliations:** Max Planck Institute for Iron Research Dusseldorf, Germany, Germany

Thank you so much for your valuable comments which were helpful in improving the manuscript. Your individual comments are addressed in italics as follows:

**Comment: **My answer to the question 'Are sufficient details provided to allow replication of the method development and its use by others?' was 'Partly'. The reason for this is because the results discussed in this paper are specific to the TWIP steel used as reference material. This fact is not mentioned in the title, the abstract or the conclusions, where the work is described as facilitating the study of hydrogen in metals, being easy to think that the work may refer to metals in general. The successful routes described in this work and the experimental parameters used here might not work in the same way for other materials (W, metallic multilayers, Zr alloys, Ti alloys, etc.) susceptible to hydrogen embrittlement as well.

***Reply:** Thanks a lot for pointing it out. In response to this comment, it has now been mentioned in the abstract and the conclusion that the study has been performed on a TWIP steel.*

**Comment: **My 'Partly' answer to this question is also due to the very specific framework (Laplace project) used to conduct the study. For somebody else willing to replicate the method, they would have to build a similar framework (not easy...), ask for access to this facility or try accessing a similar facility somewhere else, for example the one described in reference [1] below or some of those mentioned in reference 26 of the manuscript.

***Reply:** Yes, that’s a fair point, that these workflows require access to similar facilities such as N _2_ glovebox, FIB equipped with cryogenic stage, Reacthub Module or a similar gas charging chamber and vacuum carry transfer suitcases. However, the infrastructure in full is described now in the literature and can be reproduced. Gas-charging chambers used to be in operation at the University of Oxford and Iowa State University, PNNL is equipped too, one is being built also at the University of Sydney, and there are plans at FAU-Erlangen. So capabilities are on the rise overall.*

**Comment: **I found the first paragraph of the Introduction a bit vague compared to the rest of the Introduction or the rest of the paper. The different statements didn't seem to be connected or, at least, that was my perception.

***Reply**: We have modified the first paragraph of the introduction to try and improve its clarity. Thanks for pointing this out.*

**Comment: **I could spot 2 or 3 minor typos and 3 reference links not displayed correctly:

 Link to reference 1 not included, can be found as in [2]

 Reference 5 not displayed correctly, might be as [3]

 Link to reference 49 not displayed, might be as [4]

***Reply:*** *Changes in those references have been made in the manuscript.*

**Comment:**The description of the thickness 't' in Figure 4 is not clear... Is it correctly located in Fig. 4(a)? The 3um and 1um values mentioned in captions (b), (c) and (d), do they correspond to different samples? or to different stages in the same sample preparation process? It is a bit confusing.

***Reply:*** *Yes, the thickness ‘t’ refers to the thickness of the Pt weld, as shown in Figure 3(a) (It is Figure 3 in the new version which was Figure 4 in the earlier version). The 3µm and 1µm values correspond to different samples. These are 2 different samples prepared with different thicknesses of Pt welds.*
